# Hepatotoxicity of anesthetic gases

**DOI:** 10.17179/excli2020-2685

**Published:** 2020-07-27

**Authors:** Wiebke Albrecht

**Affiliations:** 1 Leibniz Research Centre for Working Environment and Human Factors

## ⁯⁯

***Dear Editor, ***

Recently, Masoud Neghab and colleagues published an article about liver enzymes in operating room personnel exposed to anesthetic gases (Neghab et al., 2020[13]). In the United States about 200,000 health care workers may be exposed to anesthetic gases (OSHA, 2000[15]). Possible adverse effects discussed in the context of exposure to anesthetic gases are hepatotoxicity (Safari et al., 2014[16]; Nicoll et al., 2012[14]; Iaizzo et al., 1990[9]) and nephrotoxicity (Jafari et al., 2018[10]). Neghab and colleagues studied 52 exposed and 52 non-exposed individuals (Neghab et al., 2020[13]). The exposed subjects showed relatively high mean urinary concentrations of 176 ppm, 5.0 ppm and 15.0 ppm nitrous oxide, isoflurane and sevoflurane (Neghab et al., 2020[13]). The authors report statistically significant increases in the liver enzymes aspartate aminotransferase, alanine aminotransferase and glutathione-S-transferase α as well as the kidney damage marker kidney injury molecule-1 compared to non-exposed individuals. It should, however, be considered that the exposure associated increase of liver enzymes was small. For example, aspartate aminotransferase increased from 19.8 ± 11.8 in non-exposed to 24.8 ± 13.2 U/L in exposed individuals (Neghab et al., 2020[13]). The corresponding activities for alanine aminotransferase were 20.8 ± 14.7 and 29.8 ± 20.7, respectively. 

Currently, numerous studies are performed to study hepatotoxicity *in vitro*, e.g. using primary human hepatocytes (Godoy et al., 2013[4]; Albrecht et al., 2019[1]; Gu et al., 2018[7]; Grinberg et al., 2014[6], 2018[5]). In animal models often the toxic solvent CCl_4_ (Hoehme et al., 2010[8]; Ghallab et al., 2016[2]) or acetaminophen (Ghallab et al., 2019[3]; Leist et al., 2017[12]) are used to study hepatotoxicity. In human liver diseases as well as in animal studies with experimental, e.g. cholestatic liver damage, a much higher increase of liver enzymes is observed (Ghallab et al., 2019[3]; Vartak et al., 2016[17]; Jansen et al., 2017[11]) compared to the present study (Neghab et al., 2020[13]). The authors of the present study (Neghab et al., 2020[13]) critically discuss if the very small increase of liver enzymes is of pathophysiological relevance or if it can be compensated without consequences. The work of Neghab and colleagues represents a valuable contribution to the long-standing question if occupational exposure to anesthetic gases causes an increased risk of hepatotoxicity. Further analyses of exposed individuals are required and a specific focus should be given to studies with a long-term follow-up to learn if operating room personnel exposed to anesthetic gases has an increased risk to develop chronic liver diseases.

## Conflict of interest

The author declares no conflict of interest.
